# HDAC1 and Klf4 interplay critically regulates human myeloid leukemia cell proliferation

**DOI:** 10.1038/cddis.2014.433

**Published:** 2014-10-23

**Authors:** Y Huang, J Chen, C Lu, J Han, G Wang, C Song, S Zhu, C Wang, G Li, J Kang, J Wang

**Affiliations:** 1 Clinical and Translational Research Center of Shanghai First Maternity and Infant Health Hospital, Shanghai Key Laboratory of Signaling and Disease Research, School of Life Science and Technology, Tongji University, 1239 Siping Road, Shanghai 200092, PR China; 2 Department of Hematology, Changhai Hospital, The Second Military Medical University, Shanghai 200433, PR China; 3 Laboratory of Population & Quantitative Genetics, Department of Biostatistics and Computational Biology, School of Life Sciences, Fudan University, Shanghai 200433, PR China

## Abstract

Acute myeloid leukemia (AML) is recognized as a complex disease of hematopoietic stem cell disorders, but its pathogenesis mechanisms, diagnosis, and treatment remain unclear. General histone deacetylase (HDAC) inhibitors have been used in blood cancers including AML, but the lack of gene specificity greatly limits their anti-cancer effects and clinical applications. Here, we found that HDAC1 expression was negatively correlated with that of Krüppel-like factor 4 (Klf4) and that AML patients with lower HDAC1 level had better prognosis. Further, knockdown of HDAC1 in leukemia cells K562, HL-60, and U937 significantly increased Klf4 expression and inhibited cell cycle progression and cell proliferation, similar results were found for HDAC inhibitors (VPA and mocetinostat). Moreover, overexpression or knockdown of Klf4 could markedly block the effects of HDAC1 overexpression or knockdown on leukemia cells *in vitro* and *in vivo*, respectively. Mechanistic analyses demonstrated that HDAC1 and Klf4 competitively bound to the promoter region of *Klf4* and oppositely regulated Klf4 expression in myeloid leukemia. We identified HDAC1 as a potential specific target for repressing cell proliferation and inducing cell cycle arrest through interplay and modulation of Klf4 expression, suggests that HDAC1 and Klf4 are potential new molecular markers and targets for clinical diagnosis, prognosis, and treatment of myeloid leukemia.

Leukemia is one of the 10 leading cancer types in terms of estimated new cancer cases and deaths in United States.^1^ In patients with leukemia, bone marrow produces abnormal white blood cells. The disease can be identified as either acute or chronic, while acute leukemia cells are very abnormal and proliferate rapidly. In children, most cases of leukemia are acute. Acute myeloid leukemia (AML) can occur in people of all ages and is a heterogeneous group of hematopoietic stem cell disorders characterized by uncontrolled proliferation of myeloblasts resulting from defects in hematopoietic differentiation, ultimately resulting in bone marrow failure.^2^ Treatments for AML include chemotherapy, radiation therapy, stem cell transplants and targeted immune therapy. However, treatment outcomes for patients with AML have not improved in the past 20 years.^3^ Further exploration for the pathogenesis mechanisms of this complex disease are needed to establish appropriate diagnosis and treatment approaches.

Recently, multiple studies have demonstrated that AML cells may exhibit several genetic and epigenetic lesions.^4^ Histone deacetylases (HDACs) are key epigenetic regulators in gene expression and cell differentiation, proliferation, apoptosis, and inflammation.^5,6^ Aberrant recruitment of HDACs is an important mechanism of gene silencing in promyelocytic leukemia.^7^ HDAC inhibitors (HDACi) can repress cell proliferation, cell cycle arrest, and cell apoptosis, which makes the prospect of HDACi treatment in humans very promising.^8,9^ Valproic acid (VPA) has been used as an antiepileptic drug for decades and has little side effects that induces malignant cell differentiation and apoptosis in a variety of cancers, including AML.^10, 11, 12^ However, HDACis, such as VPA, are broad-spectrum inhibitors and lack gene specificity, resulting in having limited anti-leukemia effects and possibly disturbing normal biological functions. Furthermore, different HDACs may modulate specific oncogenes and/or tumor-suppressor genes in different human diseases. Therefore, it will be of value to determine which specific HDAC is responsible for the anti-leukemia effects of HDACi and to explore the protein's biological function and mechanism in AML.

Krüppel-like factor 4 (Klf4) is a zinc finger transcription factor expressed in a wide variety of tissues, including gut, thymus, cardiac myocytes, and lymphocytes.^13,14^ Klf4 is important for many different physiological processes, including cell development, stem cell self-renewal, and maintenance of normal tissue homeostasis. Klf4 can function as a tumor-suppressor gene and oncogene, depending on the cellular context. Recently, Klf4 has been identified as a tumor-suppressor gene in colon, bladder, and gastric cancers.^15, 16, 17, 18^ Other studies have demonstrated that Klf4 is an oncogene in breast cancer and skin carcinoma.^19,20^ As a novel anti-hypertrophic transcriptional regulator, Klf4 mediates the HDACi-induced prevention of cardiac hypertrophy.^21,22^ Activated HDAC2 triggers hypertrophy by inhibiting the signal cascades of either Klf4 or inositol polyphosphate-5-phosphatase f (Inpp5f), indicating that Klf4 might be a specific target of certain HDACs. Recently, evidence has suggested that Klf4 may serve as a tumor suppressor in leukemia.^23,24^ Previous studies have demonstrated that Klf4 is repressed by CDX2 in AML and colon cancer,^25^ and downregulation of Klf4 by Jak2 allows for increased proliferation of progenitor cells.^26^ However, the exact role of Klf4 and HDACs in leukemia, particularly in AML, is unclear.

By evaluating the effects of specific HDACs in human myeloid leukemia, we first found that the expression level of HDAC1 was negatively correlated with Klf4 expression and that patients with lower HDAC1 levels showed a better prognosis. Further functional and mechanistic studies indicated that HDAC1 specifically targets *Klf4* and that this interplay inhibits myeloid leukemia cell proliferation and cell cycle. These results suggest that HDAC1 and Klf4 are potential molecular markers and targets for the clinical diagnosis, prognosis, and treatment of myeloid leukemia.

## Results

### Negative correlation of HDAC1 and Klf4 is significantly associated with human leukemia patients

**Figure 1:**
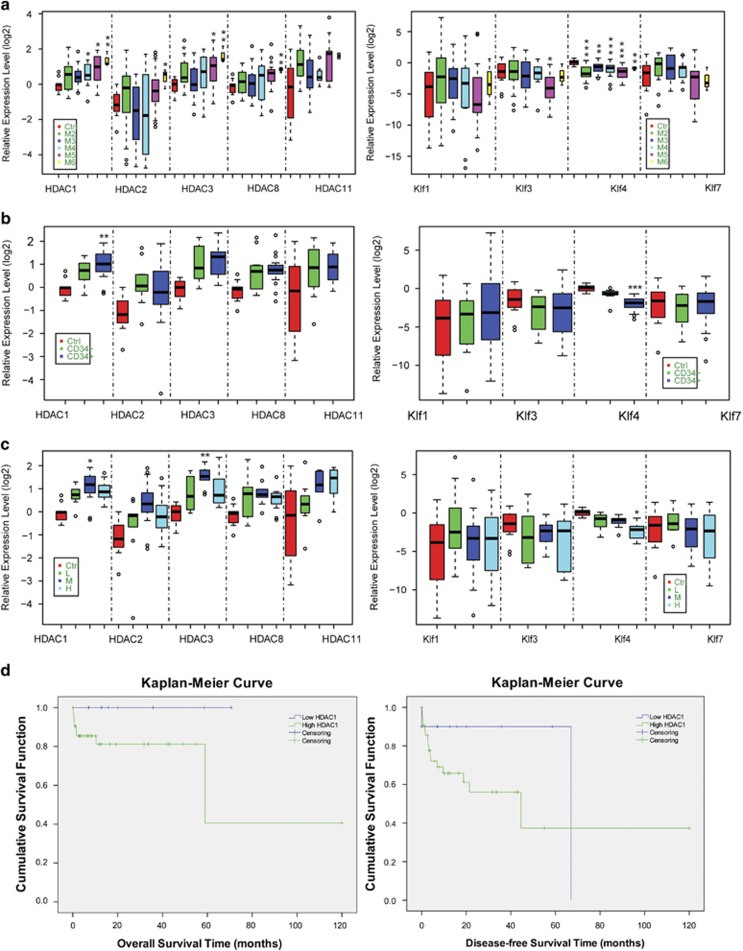
The expression levels of HDAC1 and Klf4 are significantly associated in human leukemia patients. (**a**) Analysis of the expression levels of Class I HDACs (HDAC1, HDAC2, HDAC3, and HDAC8), HDAC11, and Klfs (Klf1, KLf3, Klf4, and Klf7) in human leukemia patients with M2, M3, M4, M5, and M6. The expression levels were detected using qRT-PCR analysis with GAPDH as the internal control and compared with normal controls. *, **, and *** indicate *P*<0.05, *P*<0.01, and *P*<0.001, respectively. (**b**) Analysis of the expression levels of Class I HDACs (HDAC1, HDAC2, HDAC3, and HDAC8), HDAC11, and Klfs (Klf1, KLf3, Klf4, and Klf7) in CD34^+^ leukemia patients compared with CD34^-^ leukemia patients. ** and *** indicate *P*<0.01 and *P*<0.001, respectively. (**c**) Analysis of the expression levels of Class I HDACs (HDAC1, HDAC2, HDAC3, and HDAC8), HDAC11, and Klfs (Klf1, KLf3, Klf4, and Klf7) in leukemia patients with different cytogenetic risks (M, intermediate risk; H, high risk) compared with low-risk (L) of leukemia patients. * and ** indicate *P*<0.05 and *P*<0.01, respectively. (**d**) The Kaplan–Meier Curve showing the effects of HDAC1 on overall survival and disease-free survival function in human leukemia patients

To investigate the significance of Class I HDACs (HDAC1, HDAC2, HDAC3, and HDAC8), HDAC11, and Klfs (Klf1, KLf3, Klf4, and Klf7) in the pathogenesis of human leukemia, we used real-time RT-PCR to evaluate the expression of HDACs and Klfs in bone marrow samples from human leukemia patients. An association analysis of clinical characteristics revealed that when compared with normal controls, HDAC expression was much higher in human leukemia patients, particularly for HDAC1 (Figure 1a). HDAC1 expression levels in the cells of leukemia patients were significantly higher than controls. HDAC expression also had a significant positive correlation with FAB type (Table 1). Additionally, we found that Klf4 was significantly downregulated in leukemia patients and more negatively correlated with FAB type (Figure 1a, Table 1). While performing association analyses of the expression levels of HDACs and Klfs with immunophenotyping (CD34^−/+^ cells) and cytogenesis results, we observed that Klf4 was significantly downregulated in CD34^+^ cells and in the high-risk group of chromosomes, whereas HDAC1 was significantly upregulated in CD34^+^ cells and in the intermediate-risk group of chromosomes (Figures 1b and c). The correlation analyses for the expression level of these HDACs and Klfs indicated that most of the HDACs and Klfs were significantly negatively correlated, especially the correlation between HDAC1 and Klf4 (Table 1). Excitingly, both analyses of overall and disease-free survival functions indicated that human leukemia patients with lower levels of HDAC1 expression had better follow-up results than those with higher levels of HDAC1 expression (Figure 1d, Supplementary Table S1). These findings indicate that the negative correlation between HDAC1 and Klf4 may have a critical role in the genesis of human leukemia.

### Functional and mechanistic analyses of HDAC1 and Klf4 in leukemia cells

**Figure 2:**
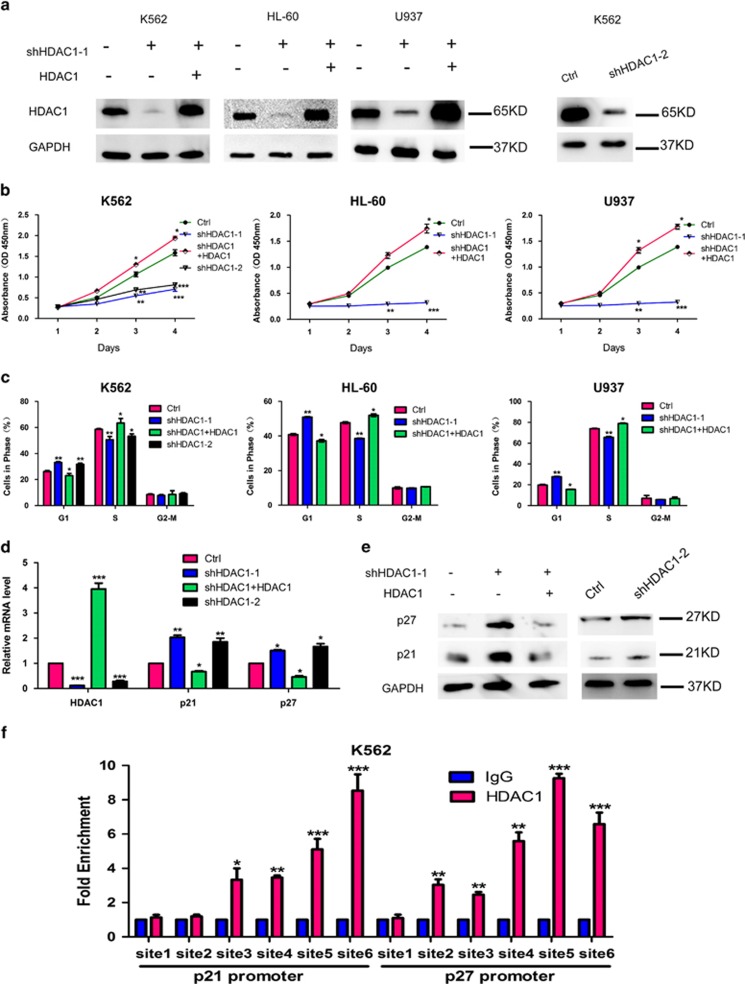
HDAC1 is critically important for proliferation of leukemia cells. (**a**) Western blotting analyses showing the knockdown efficiency of HDAC1 in the human leukemia cell lines K562, HL-60, and U937 after being infected with lentiviruses containing shRNA (shHDAC1) and the expression levels of HDAC1 after cells were co-infected with viruses containing shHDAC1 and HDAC1 (shHDAC1+HDAC1). GAPDH serves as a loading control. (**b**) The CCK8 assays showing the effects of shHDAC1 and shHDAC1+HDAC1 on cell proliferation in K562, HL-60, and U937 cells at day 1, 2, 3, and 4, respectively. *, **, and *** indicate *P*<0.05, *P*<0.01, and *P*<0.001, respectively. (**c**) FACS analyses showing the effects of shHDAC1 and shHDAC1+HDAC1 on the cell cycle in K562, HL-60, and U937 cells. * and ** indicate *P*<0.05 and *P*<0.01, respectively. (**d**) QRT-PCR assays showing the effects of shHDAC1 and shHDAC1+HDAC1 on the expression levels of HDAC1, p21, and p27 in K562 cells. GAPDH was used as the internal control. *, **, and *** indicate *P*<0.05, *P*<0.01, and *P*<0.001, respectively. (**e**) Western blotting assays showing the effects of shHDAC1 and shHDAC1+HDAC1 on the expression levels of p21 and p27 in K562 cells. Ctrl, cells infected with viruses carrying a non-targeting control vector. GAPDH was used as the loading control. *, **, and *** indicate *P*<0.05, *P*<0.01, and *P*<0.001, respectively. (**f**) ChIP-PCR assays of the binding sites of HDAC1 at the promoter regions of *p21* and *p27*, according to fold enrichment normalized to normal IgG. ** and *** indicate *P*<0.01 and *P*<0.001, respectively

To illustrate the exact function of HDAC1 in human leukemia, we first modulated the expression level of HDAC1 in the human leukemia cell lines K562, HL-60, and U937 (Figure 2a). Results showed that knockdown of HDAC1 significantly inhibited cell proliferation (Figure 2b) and induced cell cycle arrest (Figure 2c). Furthermore, knockdown of HDAC1 increased the mRNA and protein levels of p21 and p27, which are two critical cell-cycle-associated genes (Figures 2d and e). This suggests that knockdown of HDAC1 is important for inhibiting cell proliferation, a finding confirmed by similar results using VPA treatment (Supplementary Figure S1A and S1D). Besides using VPA, we also used mocetinostat that preferentially inhibits HDAC1 *versus* tubastatin A that inhibits HDAC6 to treat K562 cells. The results showed mocetinostat exhibits a stronger proliferation inhibition than tubastatin A (Supplementary Figure S2A and S2B), indicating that the effects are dependent of HDAC1. Moreover, we analyzed the mRNA and protein expression levels of p21, p27, and Klf4 in K562 cells and found that p21, p27, and Klf4 were obviously elevated after treatment with mocetinostat than with tubastatin A (Supplementary Figure S2C and S2D). In addition, the overexpression of HDAC1 could reverse the effects of HDAC1 depletion on cell proliferation, cell cycle, and the expression level of p21 and p27 (Figures 2a–e). Mechanistic studies also indicated that HDAC1 could bind to the *p21* and *p27* promoter and inhibited gene transcription (Figure 2f). These results suggest that HDAC1 promotes excessive proliferation of leukemia cells mainly by accelerating the cell cycle and repressing p21 and p27 expression.

To explore the role of other HDACs in AML cells, we used shRNAs that target HDAC2, HDAC3, and HDAC8 to treat K562 cells (Supplementary Figure S3A). The results showed that HDAC2 knockdown mainly induces apoptosis of leukemia cells, whereas HDAC3 and HDAC8 have no effect on the proliferation of leukemia cells, which are different from HDAC1 function (Supplementary Figure S3B and S3D).

**Figure 3:**
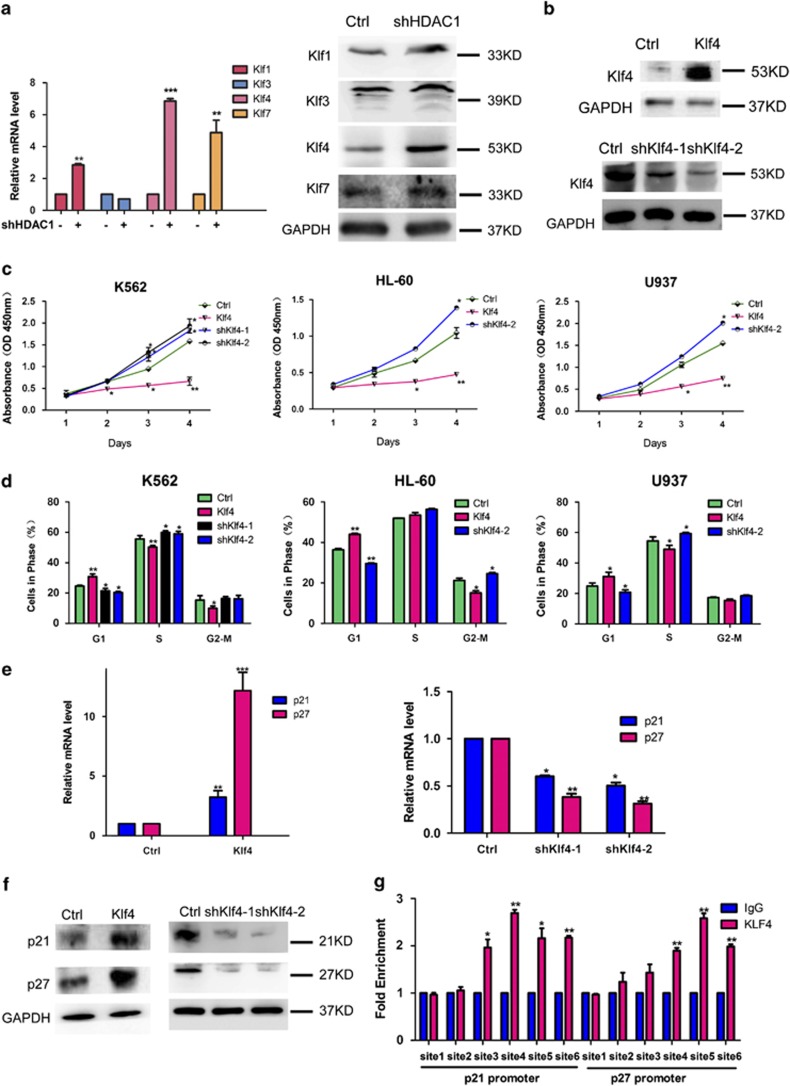
HDAC1 knockdown-induced Klf4 activation inhibits cell proliferation through upregulation of p21 and p27. (**a**) QRT-PCR analyses (left panel) and western blotting assays (right panel) showing the effects of HDAC1 knockdown (shHDAC1) on the expression of Klf1, Klf3, Klf4, and Klf7 in K562 cells. ** and *** indicate *P*<0.01 and *P*<0.001, respectively. (**b**) Overexpression and knockdown efficiency of Klf4 after being infected with lentiviruses containing the cDNA (Klf4) and shRNA (shKlf4) for 72 h in K562 cells. GAPDH was used as a loading control. (**c**) CCK8 assays showing the effects of overexpression and knockdown of Klf4 on cell proliferation in K562, HL-60, and U937 cells at day 1, 2, 3, and 4, respectively. * and ** indicate *P*<0.05 and *P*<0.01, respectively. (**d**) Analyses of the effects of overexpression and knockdown of Klf4 on cell cycle in K562, HL-60, and U937 cells. Cells were collected and fixed using 75% ethanol, stained using PI and measured using FACS assay. * and ** indicate *P*<0.05 and *P*<0.01, respectively. (**e**) QRT-PCR analyses showing the effects of overexpression and knockdown of Klf4 on the expression levels of p21 and p27. GAPDH was used as the internal control. *, **, and *** indicate *P*<0.05, *P*<0.01, and *P*<0.001, respectively. (**f**) Western blotting assays showing the effects of overexpression and knockdown of Klf4 on the expression levels of p21 and p27. GAPDH was used as the loading control. (**g**) ChIP-PCR assays of the binding sites of Klf4 at the promoter regions of *p21* and *p27*, according to fold enrichment normalized to normal IgG. *, **, and *** indicate *P*<0.05, *P*<0.01, and *P*<0.001, respectively

**Figure 4:**
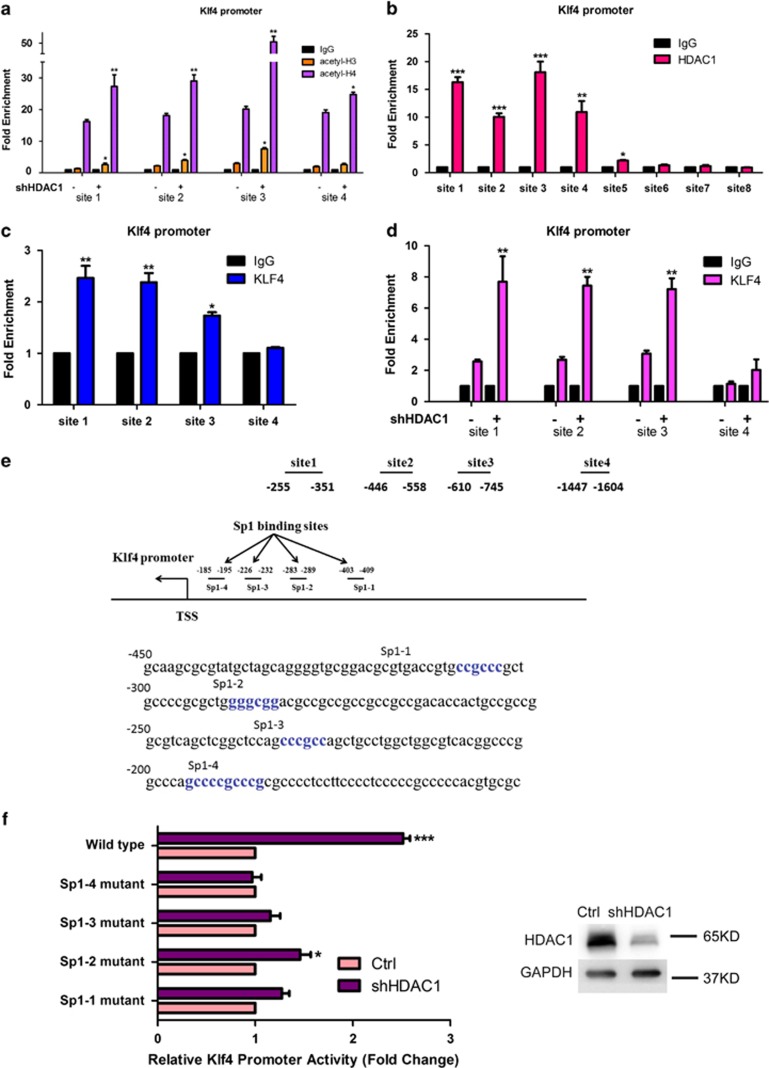
HDAC1 affects *Klf4* promoter activity through Sp1-binding domains. (**a**) ChIP-PCR assays of acetylation levels of histone H3 and H4 at the *Klf4* promoter regions after knockdown of HDAC1 (shHDAC1) in K562 cells. * and ** indicate *P*<0.05 and *P*<0.01, respectively. (**b**) ChIP-PCR assays of HDAC1 binding at *Klf4* promoter regions in K562 cells. *, **, and *** indicate *P*<0.05, *P*<0.01, and *P*<0.001, respectively. (**c**) ChIP-PCR assays of Klf4 binding at its own promoter regions in K562 cells. * and ** indicate *P*<0.05 and *P*<0.01, respectively. (**d**) ChIP-PCR assays of Klf4 binding at its own promoter after knockdown of HDAC1 in K562 cells. ** indicates *P*<0.01. (**e**) Site1, site2, site3, and site4 of *Klf4* promoter located between −255 and −351, −446 and −556, −610 and −745, and −1447 and −1604 from the transcription starting site, respectively. Four Sp1-binding elements are underlined and labeled as Sp1-1, Sp1-2, Sp1-3, and Sp1-4. (**f**) Dual-luciferase assay studied in HEK293T cells transfected with wild-type Klf4 promoter plasmid, Sp1-1 mutant plasmid, Sp1-2 mutant plasmid, Sp1-3 mutant plasmid, and Sp1-4 mutant plasmid. * and *** indicate *P*<0.05 and *P*<0.001, respectively

In consistent with the result of clinical analyses, knockdown of HDAC1 significantly increased the expression of Klfs, particularly Klf4 (Figure 3a). Again, different from HDAC1 knockdown, knockdown of HDAC2, HDAC3, and HDAC8 showed no significant effect on Klf4 expression (Supplementary Figure S3E). Furthermore, using HDACis VPA and mocetinostat, we found that Klf4 is elevated in HDACi-treated cells (Supplementary Figure S1D, S2D, S4A, and S4B). These data demonstrated HDAC1 is negatively correlated with Klf4. Modulation of Klf4 levels by altering shRNA or using an ectopic expression vector can mimic the effects of HDAC1 on cell proliferation, cell cycle, and the expression level of p21 and p27 in human leukemia cell lines K562, HL-60, and U937 (Figures 3b and f). Furthermore, chromatin immunoprecipitation (ChIP)-PCR assays showed that Klf4 can bind to the promoter regions of both *p21* and *p27* (Figure 3g). Primers used in this assay targets *p21* and *p27* promoter at six sites (Supplementary Figure S4C). Dual-luciferase reporter gene assay also confirmed that Klf4 could bind at the promoter regions of *p27* (Supplementary Figure S4D). All of these results indicate that HDAC1 knockdown inhibits cell proliferation mainly through Klf4 activation, direct binding of Klf4 to *p21* and *p27* promoter regions, and the induction of cell cycle arrest. Mechanistic studies also indicate that knockdown of HDAC1 increased histone acetylation levels at the *Klf4* promoter region and that both HDAC1 and Klf4 can bind to the *Klf4* promoter (Figures 4a–c) without direct interaction at the protein level (Supplementary Figure S4E). Cells treated with VPA also showed significantly higher acetylation levels of histone H3 and H4 at the *Klf4* promoter region (Supplementary Figure S4F). Furthermore, HDAC1 knockdown also enhanced the binding of Klf4 at its own promoter regions (Figure 4d), which indicates that HDAC1 and Klf4 may compete in binding at the *Klf4* promoter region. Primers used in this assay targets *Klf4* promoter at four sites (Figure 4e): site 1 (−255, −351), site 2 (−446, −558), site 3 (−610, −745), and site 4 (−1447, −1604). The information of other four sites was included in Supplementary Table S2. Four binding elements of stimulatory protein (Sp1) were indentified on the proximal portion of the *Klf4* promoter and were named as Sp1-1, Sp1-2, Sp1-3, and Sp1-4 (Figure 4e). Mutation of the Sp1-1, Sp1-3, or Sp1-4 site significantly killed HDAC1 knockdown-stimulated *Klf4* promoter activity whereas Sp1-2 decreased part of the activity of *Klf4* promoter (Figure 4f). Data in Supplementary Figure S4G also demonstrated that HDAC1 can mediate the luciferase activities of *Klf4* promoter. These studies suggested that transactivation of Klf4 by HDAC1 knockdown or VPA appeared to be mediated through interaction with the Sp1-binding domain on the promoter and is also likely to involve histone acetylation. To investigate the role of Sp1 in Klf4 transcriptional regulation, we performed western blotting analyses to detect the expression and acetylation level of Sp1 upon mocetinostat treatment. Results showed that Sp1 expression was reduced and acetyl-Sp1 level was elevated upon mocetinostat treatment (Supplementary Figure S4H and S4I). Furthermore, mocetinostat repressed the binding of Sp1 at *Klf4* promoter (Supplementary Figure S4J). These data indicated that Sp1 may interact with HDAC1 to repress Klf4 transcription, whereas HDACi would repress Sp1-mediated inhibition of Klf4 expression.

**Figure 5:**
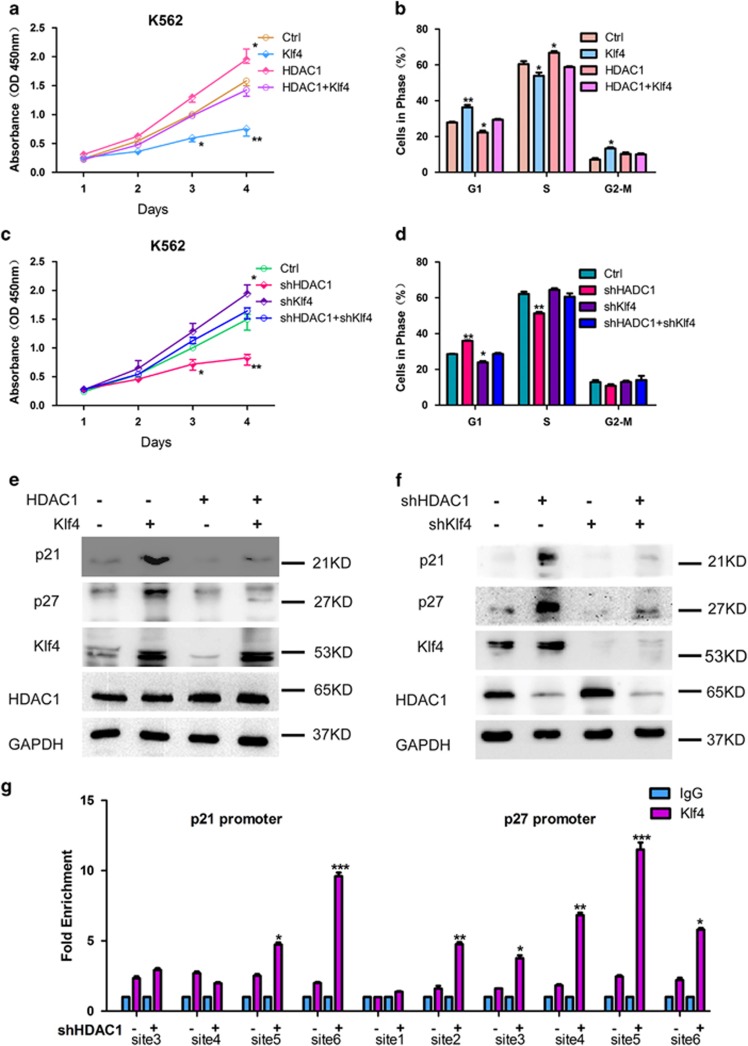
Klf4 significantly rescues the effects of HDAC1 on leukemia cell proliferation. (**a**) Effects of ectopic expression of Klf4 on HDAC1-induced cell proliferation. * and ** indicate *P*<0.05 and *P*<0.01, respectively. (**b**) FACS analysis showing the effects of ectopic expression of Klf4 on HDAC1-induced cell cycle. * and ** indicate *P*<0.05 and *P*<0.01, respectively. (**c**) CCK8 analysis showing the effects of Klf4 knockdown on HDAC1 deficiency-mediated cell proliferation. * and ** indicate *P*<0.05 and *P*<0.01, respectively. (**d**) FACS analysis showing effects of Klf4 knockdown on HDAC1 deficiency-mediated cell cycle. * and ** indicate *P*<0.05 and *P*<0.01, respectively. (**e**) Western blotting analyses showing the effects of ectopic expression of Klf4 and HDAC1 on the expression levels of p21 and p27. GAPDH was used as the loading control. (**f**) Western blotting analyses showing the effects Klf4 and HDAC1 knockdown on the expression levels of p21 and p27. GAPDH was used as the loading control. (**g**) ChIP-PCR assays showing Klf4 binding at the promoter region of *p21* and *p27* after HDAC1 knockdown. Control or HDAC1-deficient cells were fixed and subjected to ChIP assay with anti-Klf4 antibody. ChIP-PCR primers were used for *p21* and *p27* promoters where shown in Supplementary Table S2. *, **, and *** indicate *P*<0.05, *P*<0.01, and *P*<0.001, respectively

To confirm these findings, we performed rescue experiments using induced overexpression of Klf4 in HDAC1-overexpressed K562 cells and Klf4 knockdown in HDAC1-inhibited K562 cells. The modulation of Klf4 expression level significantly reversed the corresponding effects of HDAC1 on cell proliferation (Figures 5a and c), the cell cycle (Figures 5b and d), and p21 and p27 expression (Figures 5e and f). Knockdown of HDAC1 consistently increased Klf4 binding at *p21* and *p27* promoter regions (Figure 5g), indicating that HDAC1 and Klf4 also compete to bind at these promoter regions.

### *In vivo* studies of the function and mechanism of HDAC1 and Klf4

**Figure 6:**
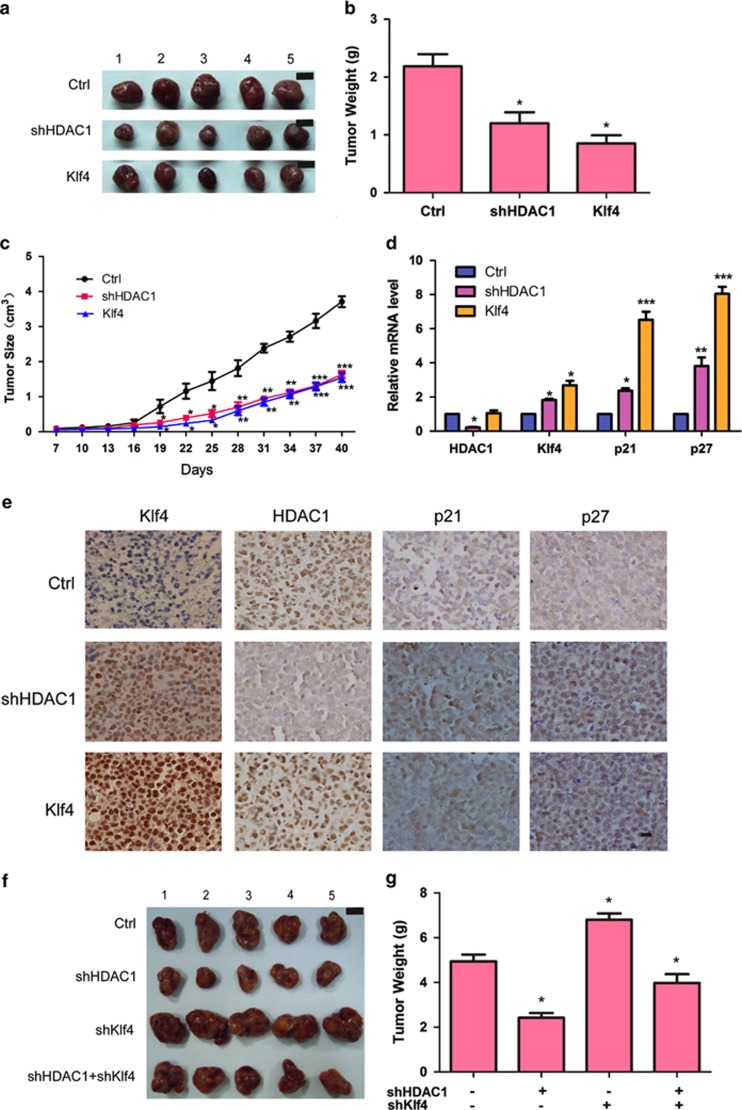
Klf4 has a critical role in HDAC1-induced leukemia cell proliferation *in vivo*. (**a**) Representative images of tumors derived from mice injected with control, HDAC1-knockdown, and Klf4-overexpressed cells, respectively. (**b**) Tumor weight after injection with control, HDAC1-knockdown, and Klf4-overexpressed cells for 6 weeks. * indicates *P*<0.05. (**c**) Tumor size observed every 3 days of mice injected with control, HDAC1-knockdown, and Klf4-overexpressed cells, respectively. *, **, and *** indicate *P*<0.05, *P*<0.01, and *P*<0.001, respectively. (**d**) QRT-PCR analyses of the expression levels of HDAC1, Klf4, p21, and p27 in tumors of the control, HDAC1-knockdown, and Klf4-overexpressed cell groups. *, **, and *** indicate *P*<0.05, *P*<0.01, and *P*<0.001, respectively. (**e**) Immunohistochemical staining of HDAC1, Klf4, p21, and p27 in tumors derived from the control, HDAC1-knockdown, and Klf4-overexpressed cell groups. Scale bar, 100 *μ*m. Images were acquired at room temperature using a Nikon 90i Eclipse microscope system (Nikon), Nikon DigiSight Digital Camera Head, and Nikon NSI-Elements Version 3.10 software. (**f**) Representative images of tumors derived from mice after injection with control, HDAC1-knockdown cells, Klf4-knockdown cells, and cells with HDAC1 and Klf4 knockdown, respectively. Scale bar, 10 mm. (**g**) Tumor weight after injection with control, HDAC1-knockdown, Klf4-knockdown cells, and HDAC1/Klf4-knockdown cells, respectively. * indicates *P*<0.05

To further investigate the function and mechanism of HDAC1 and Klf4 in leukemia cells, we performed tumorigenesis experiments in BLAB/c nude mice. Results showed that the tumor weights and growth rates of mice injected with Klf4-overexpressed or HDAC1-inhibited cells were significantly lower than those of the control group (Figures 6a–c). We also found that HDAC1 knockdown or Klf4 overexpression significantly increased p21 and p27 expression (Figures 6d and e) in tumors. The tumor proliferation and tumor size was consistent with *in vitro* studies and was confirmed by the *in vivo* rescue experiment (Figures 6f and g). The *in vivo* tumorigenesis results provide more evidences that the inhibition of HDAC1, overexpression of Klf4, or a combination of the two may provide a novel way to treat leukemia in humans.

## Discussion

AML is a complex disease of hematopoietic stem cell disorders, and its pathogenesis mechanisms, diagnosis, and treatment require further study.^1^ Epigenetic inhibitors such as HDACi are widely used in clinicals,^11^ but HDACis including VPA, mocetinostat, and tubastatin A, have side effects, limiting their anti-cancer effects. In this study, expression levels of HDACs were significantly higher in AML patients than in normal controls, whereas lower HDAC1 expression predicted a better prognosis for those patients by overall and disease-free survival time assay.

It is well-known that HDACis, a novel and promising class of chemotherapeutic agents, can induce cell cycle arrest, apoptosis, and differentiation. VPA, mocetinostat, and tubastatin A, which are inhibitors of Class I and II HDASs, have been reported with strong anti-cancer activity in a variety of human cancers.^10, 11, 12^ However, it is unclear which HDAC is the most important target for treating and understanding various cancers. We have previously reported that VPA inhibits breast cancer cell migration by specifically targeting HDAC2 and down-regulating survivin.^27^ In this study, we found that HDAC1 and HDAC2 had an important role in myeloid leukemia cells. Knockdown of HDAC1 can mimic the effects of VPA and mocetinostat by inducing cell cycle arrest and inhibiting leukemia cell proliferation. Knockdown of HDAC2 induces apoptosis of leukemia cells, which is different from HDAC1 knockdown. Further studies suggested that the mechanism by which HDAC1 affects myeloid leukemia cells is mainly by regulating Klf4.

Previous evidences have suggested that Klf4, which is a zinc finger transcription factor, can function as both a tumor-suppressor gene and an oncogene and might be specifically targeted by certain HDACs.^15, 16, 17, 18, 19, 20^ Consistent with our study, Klf4 has been recently shown to act as a tumor suppressor in Hodgkin lymphoma,^23,28^ but the exact mechanism by which Klf4 affects myeloid leukemia remains unclear. In this study, we found that expression of Klf4 was significantly lower in AML patients than in normal controls, consistent with previous findings.^29^ Furthermore, we identified that forced expression of Klf4 significantly inhibited cell proliferation by inducing cell cycle arrest and increasing the expression of p21 and p27 *in vitro* and *in vivo*. These findings suggest that Klf4 may be a new predictive, diagnostic, and treatment target for AML patients.

Previous studies have reported that Klf4 signal cascades may be a target for certain HDACs during HDACi-induced prevention of cardiac hypertrophy.^21,22^ Coincidentally, we found that knockdown of HDAC1 increased the accumulation of acetylated histone H3 and H4 in the promoter region of *Klf4*, which facilitated the binding of Klf4 to its own promoter and upregulated its expression. Further analyses demonstrated that HDAC1 and Klf4 can competitively bind to the promoter region of *Klf4*, providing a new regulation relationship between HDAC1 and Klf4. Previous studies have reported that butyrate and trichostatin A interact with a Sp1-binding site at the *Klf4* promoter to transactivate *Klf4* promoter activity.^30^ Using wild-type and mutant constructs of the *Klf4* promoter, we showed that mutation in Sp1-binding sites killed the activity of Klf4 promoter induced by HDAC1 knockdown. We can speculate that HDAC1-responsive element was located at Sp1-binding sites. Sp1 may recruit HDAC1 altering histone acetylation level at *Klf4* promoter and affecting Klf4 transcription. Once Sp1 and HDAC1 do not exist, Klf4 may bind to the *Klf4* promoter and initiate Klf4 transcription. In prostate cancer, HDACis can stimulate H3 methylation and upregulate Klf4 expression *via* Sp1 downregulation. Huang *et al.*^31^ have reported that H3 methylation can antagonize chromatin folding benefiting transcription factors binding and transcription initiation, as histone acetylation does. It suggests a crosstalk mechanism between histone acetylation and H3K4 demethylation, which underlies the complexity of the functional role of HDACs in the regulation of histone modifications. In our study, modulation of Klf4 expression can mimic the effects of HDAC1 in leukemia cells. These findings provide new evidence for understanding the pathogenesis of AML and the relationship between HDAC1 and Klf4. This study also found that patients with lower HDAC1 and higher Klf4 levels had better prognosis, making these proteins new potential diagnostic and prognostic markers and therapy targets for myeloid leukemia.

## Materials and Methods

### Materials

The VPA and doxycycline used in the study were obtained from Sigma (Sigma-Aldrich, St. Louis, MO, USA). Mocetinostat and tubastatin A were purchased from Selleck (Houston, TX, USA). The pGL3-promoter luciferase plasmid and Fuw-tetO-hKlf4 were purchased from Addgene (Cambridge, MA, USA). The target sequences of the shRNA were as follows: shHDAC1-1, AACTATGGTCTCTACCG AAAA; shHDAC1-2, AACCGGTCATGTCCAAAGTAA; shKlf4-1, GAGTTCCCATCTCA AGGCACACC; shKlf4-2, GATCAAGCAGGAGGCGGT CTC, shHDAC2, ACTGCATA TTAGTCCTTCATT; shHDAC3, AAGGAGCTTCCCTATAGTGAA; shHDAC8, GGGAATA TTACGATTGCGACG. The human Flag-HDAC1 overexpression vector was generated with primers as ATCCACCGGTATGGATTACAAGGATGACGACGATAAGATGGCGCA GACGCA GGGCACCCGGA (Forward) and ATCCGAATTCTCAGGCCAACTTGACC TCCTCCTTG (Reverse). The corresponding mutant vectors were obtained by using QuickChange Lightning Multi Site Directed Mutagenesis Kit (Agilent Technologies, Santa Clara, CA, USA; Cat#210515-5). All transfections were performed using X-tremeGENE HP (Roche, F. Hoffmann-La Roche Ltd, Basel, Switzerland) according to the manufacturer's instructions. The primary antibodies used were p21 (Abcam, Cambridge, MA, USA, ab7960), p27 (Bioworld, St. Louis Park, MN, USA; BS3714), Klf4 (Santa Cruz, Santa Cruz, CA, USA; sc-20691), GAPDH (Santa Cruz, sc-166574), Acetyl-histone H3 (Millipore, Billerica, MA, USA; 06-599), Acetyl-histone H4 (Millipore, 06-598), HDAC1 (Sigma, St. Louis, MO, USA; SAB4501382), HDAC2 (Santa Cruz, sc-7899), HDAC3 (BD Transduction Laboratories, San Jose, CA, USA; 611124), HDAC8 (Santa Cruz, sc-17778), Sp1 (Millipore, 07-645), Acetylated-Lysine Antibody (Cell Signaling Technology, Danvers, MA, USA; 9441), KLF1 (Bioworld, BS2303), KLF3 (Bioworld, BA2802-2), KLF7 (Bioworld, B125-ap), and the human Klf4 antibody (AF3640) and normal Goat IgG (AB-108-C) for the ChIP assay was purchased from R&D systems (Minneapolis, MN, USA). The secondary antibodies for rabbit (Cell Signaling Technology, 7074S) or mouse (Cell Signaling Technology, 7076S), and normal rabbit IgG (Cell Signaling Technology, 9002S) or mouse IgG (Millipore, 200601) were used as internal controls.

### Cells and patients

The human leukemia cell lines K562, U937, and HL-60 were purchased from American Type Culture Collection (Manassas, VA, USA) and were cultured in RPMI-1640 (Hyclone, Logan, UT, USA) medium supplemented with 10% fetal bovine serum (Gibco, Carlsbad, CA, USA). The HEK293T cell line used for lenti-virus production was supported with high-glucose DMEM (Gibco) medium containing 10% fetal bovine serum. The virus suspension was collected and purified at 48 h after transfection. All cells were maintained at 37 °C with 5% CO_2_. A total of 74 bone marrow samples from human leukemia patients and 15 bone marrow samples taken as normal controls were collected from Changhai Hospital in Shanghai with written consent. The clinical characteristics of these samples are summarized in Supplementary Table S3.

### Quantitative RT-PCR

Total RNA was extracted from leukemia cells using RNAiso plus (TaKaRa BIO Inc, Otsu, Japan), and the first strand cDNA synthesis was performed using a TIANScript RT Kit (TIANGEN, Beijing, China) and Oligo(dT)_15_ primer from 2 *μ*g total RNA according to the manufacturer's instructions. The mRNA levels were observed using quantitative PCR performed with *SYBR Premix Ex Taq* *II* (TaKaRa BIO Inc) on a Mx3000P QPCR System (Agilent Technologies). The relative expression level of each candidate gene was calculated using GAPDH as the internal normalized control with the same calibrator. Each experiment was performed independently in triplicate. Primers for qRT-PCR are listed in Supplementary Table S4.

### Western blotting

Cells were collected, washed twice and suspended in lysis buffer. Cell lysates were prepared and analyzed using sodium dodecyl sulfate-polyacrylamide gel electrophoresis and were transferred to nitrocellulose membranes (Whatman, Dassel, Germany). Bands were visualized using an enhanced chemiluminescence system.

### Cell proliferation assay

For cell proliferation analysis, leukemia cells were seeded at a density of 3500 cells per well in 96-well plates. The OD value was measured using a microplate reader after the addition of CCK8 (Dojindo, Kumamoto, Japan) for 1 h.

### Flow cytometry assay

In preparation for cell cycle distribution analysis, 1 × 10^6^ cells were fixed with 70% ethanol and stained with PI (Sigma). These cells were then subjected to fluorescence-activated cell sorting using an FACS Calibur (BD Biosciences, San Jose, CA, USA), and data analyzed using ModFit LT cell-cycle analysis software (Verity Software House, Topsham, ME, USA).

### ChIP assay

ChIP assays were performed according to a previously established protocol.^32^ Chromatin-associated proteins were immunoprecipitated with specific antibodies, after which the DNA was purified, suspended in TE buffer and analyzed using SYBR Green fluorescence dye. The ChIP-PCR primers for *p21*, *p27*, and *Klf4* promoter are listed in Supplementary Table S2.

### Luciferase reporter assay

The promoter region (2000 bp) of human *Klf4* promoter was amplified using PrimerSTAR HS DNA Polymerase with GC buffer (TaKaRa BIO Inc, DR010GC) and cloned into pGL3-vector with double-digestion of *Kpn*I and *Hind*III. The PCR primers for *hKlf4* are as follows: 5′-ATCCGGTACCACTTGA AGTTTCTTGCTTCTTTTAG-3′ (Forward); 5′-ATCCAAGCTTACGCAAAAATAGACAAT CAGCAAGG-3′ (Reverse). Sp1-1 mutant, Sp1-2 mutant, Sp1-3 mutant, and Sp1-4 mutant vectors were generated with deletion mutation by using QuickChange Lightning Multi Site Directed Mutagenesis Kit (Agilent Technologies, Cat#210515-5). The *p27* promoter carrying sequences of −2058 to −708 and −694 to +200 were inserted into pGL-3 vector, and labeled as *p27* promoter 1-Luci and *p27* promoter 2-Luci, respectively. *Klf4* promoter carrying sequences of −1694 to −903 and −820 to +153 were inserted into pGL-3 vector, and labeled as *Klf4* promoter 1-Luci and *Klf4* promoter 2-Luci, respectively. Primers for constructing these plasmids are listed in Supplementary Table S5. HEK293T cells post-transfected with pGL3-*Klf4* promoter or pGL3-*p27* promoter and Renilla luciferase for 48 h were harvested and analyzed using the Dual-luciferase reporter system (Promega, Madison, WI, USA) according to the manufacturer's recommendations. The assay was performed in three independent experiments.

### *In vivo* tumorigenesis

In preparation for tumorigenesis, 1 × 10^7^ cells of K562 infected with viruses for 3 days were injected into the backs of 5-week-old BLAB/c nude mice raised in experimental animal center of Tongji University and each group contained five mice. Doxycycline was administered in water every 4 days at a final concentration of 1 mg/l. Tumor volumes were measured by caliper every 2 days. All experiments were carried out as approved by the Institutional Animal Care and Use Committee of Tongji University.

### Statistical analysis

All experiments were performed in triplicates. Data are shown as the mean±S.D., and analyzed with Student's *t*-test. The Wilcox rank sum and Log-Rank tests were used for Figure 1. *P*<0.05 was considered statistically significant.

## Figures and Tables

**Table 1:**
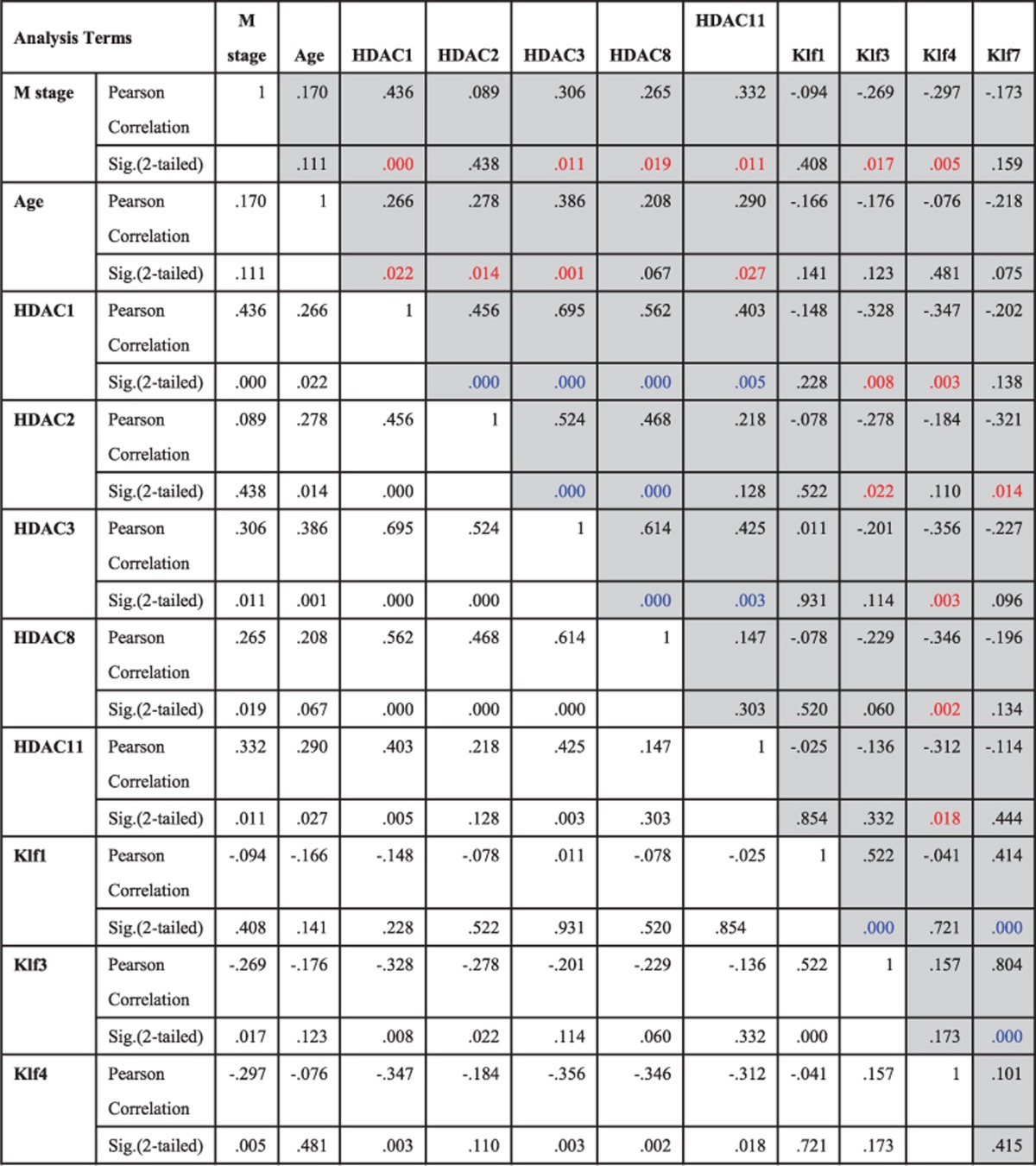
Pearson correlation analysis for the expression level of HDAC11, Class I HDACs and Klf1, Klf3, Klf4, Klf7 in leukemia patients

## Abbreviations

AML: Acute myeloid leukemia
Sp1: Stimulatory protein 1
HDAC: Histone deacetylase
Klf4: Krüppel-like factor 4
CCK8: Cell Counting Kit-8
p21: Cyclin-dependent kinase inhibitor 1A
p27: Cyclin-dependent kinase inhibitor 1B
VPA: Sodium valproic acid
ChIP: Chromatin immunoprecipitation
