# Supplementary material for: HDAC1 and Klf4 interplay critically regulates human myeloid leukemia cell proliferation
Source: Cell Death Dis. 2014 Oct 23;5(10):e1491–. doi: 10.1038/cddis.2014.433 (PMC4237257; doi:10.1038/cddis.2014.433)
Supplement: Supplementary Information [file cddis2014433x1.doc]

**Supplemental Information**

**Supplemental Tables**

**Table S1. Cox regression analysis for the expression level of Class I HDACs, Klf1, Klf3, Klf4, Klf7 and survival time in leukemia patients**

| **Cox’s Proportional Hazard Model** | | **B** | **SE** | **Wald** | **df** | **Sig. (2-tailed)** | **Exp (B)** |
| --- | --- | --- | --- | --- | --- | --- | --- |
| **HDAC1** | Overall Survival Time | 1.054 | .523 | 4.062 | 1 | .044 | 2.869 |
|  | Disease-free Time | .871 | .344 | 6.405 | 1 | .011 | 2.389 |
| **HDAC2** | Overall Survival Time | .394 | .204 | 3.715 | 1 | .054 | 1.483 |
|  | Disease-free Time | .357 | .134 | 7.048 | 1 | .008 | 1.429 |
| **HDAC3** | Overall Survival Time | .622 | .411 | 2.284 | 1 | .131 | 1.862 |
|  | Disease-free Time | .775 | .267 | 8.433 | 1 | .004 | 2.171 |
| **HDAC8** | Overall Survival Time | .560 | .387 | 2.092 | 1 | .148 | 1.750 |
|  | Disease-free Time | .566 | .264 | 4.599 | 1 | .032 | 1.761 |
| **Klf1** | Overall Survival Time | -.022 | .068 | .108 | 1 | .742 | .978 |
|  | Disease-free Time | -.023 | .043 | .286 | 1 | .593 | .977 |
| **Klf3** | Overall Survival Time | -.076 | .137 | .312 | 1 | .576 | .926 |
|  | Disease-free Time | -.136 | .091 | 2.237 | 1 | .135 | .872 |
| **Klf4** | Overall Survival Time | -.011 | .325 | .001 | 1 | .972 | .989 |
|  | Disease-free Time | .085 | .214 | .159 | 1 | .690 | 1.089 |
| **Klf7** | Overall Survival Time | -.017 | .145 | .013 | 1 | .908 | .983 |
|  | Disease-free Time | -.129 | .097 | 1.763 | 1 | .184 | .879 |

**Table S2**. Primer sequences for quantitative ChIP-PCR assay

| **Gene promoter** | **Forward** | **Reverse** |
| --- | --- | --- |
| Klf4 pro-1(site1) | GTGAGTACGGCCCTGGTCGCGCAGC | GCAGTGGTGTCGGCGGCGGCGGCGG |
| Klf4 pro-2(site2) | CGCTGAGCGACGAGAGCGGACTCCT | TGGAGAAGAGCGCGATTATCCGCGT |
| Klf4 pro-3(site3) | TGCCTCTTTCCGCCTGTT | GACCTCGCACGGTTCCTC |
| Klf4 pro-4(site4) | TGATCACAAACCAAGGGG | GTGACCATGTGCCAGGAA |
| Klf4 pro-5(site5) | GAGGGCCATAACCTTGTTGTCATAC | TTCGTGGCAGGCAACTAGCTGGTTA |
| (-2905,-2776) |  |  |
| Klf4 pro-6(site6) | CTTAGAGAAATAAAAGTAAAGCAGA | CCCTGGATGAACAGTGGATGAGCCC |
| (-2566,-2432) |  |  |
| Klf4 pro-7(site7) | ATGTTGCCATCACAATTAAAGGAGT | CTATATAGGATCAGGCCAGTTACTT |
| (-2362,-2238) |  |  |
| Klf4 pro-8(site8) | ACTCTGGATCTTAGAGGGATTCCTG | TTAGGTTTCCTCAGAATATTTGTGA |
| (-2095,-1955) |  |  |
| p21 pro-1(site1) | TATGAGAGTCCTTGTGGGCCT | TGATGCTAGGAACATGAGCAA |
| p21 pro-2(site2) | AGTAGCTGGGATTACAGGCAT | AACAGGGTATGTGATCTGCCA |
| p21 pro-3(site3) | TCCATCAAGGCTTCTGCAAAT | TAACAACATCCCCCAGCTTGT |
| p21 pro-4(site4) | TGATGCTAGGAACATGAGCAA | AGTAGCTGGGATTACAGGCAT |
| p21 pro-5(site5) | AACAGGGTATGTGATCTGCCA | TTCTGCCCTGAAAAAGGCAA |
| p21 pro-6(site6) | TGTGAAGCTCAGTACCACAAAAA | AATGTCCAGCAGAGGACAGG |
| p27 pro-1(site1) | AAAACTAGTTCTGGGCACTG | GCTTGTTTTCTTAGCCACAT |
| p27 pro-2(site2) | AGTCTCGATTTCCTCCGTG | GTGCCTACCTCATCTCATA |
| p27 pro-3(site3) | GTCCCTTCCAGCTGTCA | GGAAACCAACCTTCCGTTCT |
| p27 pro-4(site4) | TTGAAGATCCACTGAGCTTT | GCGAACGTCTTTCTTTTAGA |
| p27 pro-5(site5) | GATAAGTGCCGCGTCTAC | GAAGTTTCTGCCATCCCT |
| p27 pro-6(site6) | AGCCAGAGCAGGTTTGTT | GACACGGCGAGTCTATTT |

**Table S3. Clinical characteristics of leukemia patients and normal controls**

| **Characteristics** | **Patients** | **Normal** |
| --- | --- | --- |
| **Gender** |  |  |
| Male | 44 | 6 |
| Female | 30 | 9 |
| **Age (years old)** |  |  |
| Range | 8-87 | 13-75 |
| Mean | 47 | 37 |
| **FAB Type** |  |  |
| M2 | 21 | / |
| M3 | 17 | / |
| M4 | 14 | / |
| M5 | 19 | / |
| M6 | 3 | / |
| **Immunophenotyping** |  |  |
| CD34^-^ | 15 | / |
| CD34^+^ | 23 | / |
| Null | 36 | / |
| **Cytogenetic Risk** |  |  |
| L | 10 | / |
| M | 14 | / |
| H | 14 | / |
| Null | 36 | / |
| **Follow-up** |  |  |
| CR | 36 | / |
| NR | 12 | / |
| Ded | 9 | / |
| Others | 17 | / |

Using immunohistochemistry, cytochemistry and molecular genetics analysis, patients were classified into M1, M2, M3, M4, M5, and M6 subtypes through FAB type. According to standards of consensuses reached by Chinese experts on the leukemia and the NCCN Clinical Practice Guidelines in Oncology, in combination with cytogenetic and molecular abnormalities, the risk status was categorized into three groups: high-risk (H), intermediate-risk (M), low-risk (L). When deciding treatment, both disease-specific and individual patient factors were taken into consideration. The NCCN Guidelines were applied and consulted in the treatment of patients.

**Table S4. Primer sequences for quantitative RT-PCR assay**

| **Gene** | **Forward** | **Reverse** |
| --- | --- | --- |
| HDAC1 | ACTGGGGACCTACGG | ACTTGGCGTGTCCTT |
| HDAC2 | ATGGCGTACAGTCAAGGAGG | TGCGGATTCTATGAGGCTTCA |
| HDAC3 | CGCCTGGCATTGACCCATAG | CTCTTGGTGAAGCCTTGCATA |
| HDAC8 | TTTGAGCGTATTCTCTACGTGGA | ACACTGTAGTACCGTCCCTTC |
| HDAC11 | GGGCATCTCCAGGGCTAC | TGGGTAGATGTGGCGGTT |
| Klf1 | AGTACCAAGGGCACTTCCAGCTCTT | TATGGCTTCTCCCCTGTGTGCGT |
| Klf2 | TTCGGTCTCTTCGACGACG | TGCGAACTCTTGGTGTAGGTC |
| Klf3 | ATGTTTGACCCAGTTCCTGTCAAGCAAGA | GCTACTACTGGAATTTTCCATCTCCTCC |
| Klf4 | CAAGTCCCGCCGCCGCTCCATTACCAA | CCACAGCCGTCCCAGTCACAGTGG |
| Klf5 | CCTGGTCCAGACAAGATGTGA | GAACTGGTCTACGACTGAGGC |
| Klf6 | GGCAACAGACCTGCCTAGAG | CTCCCGAGCCAGAATGATTTT |
| Klf7 | GGTGAGCCAGACAGACTGACAA | GAAGTAGCCGGTGTCGTGGA |
| Klf8 | ATGGATAAACTCATAAACAACTTGGAGG | TCACATGGTGTCATGGCGACGGCGATGC |
| Klf11 | TCTGACTCTGGGGATGTCAC | CGGCAATCTGGAGTCTGGA |
| Klf13 | ATGGCAGCCGCCGCCTATGTGGACCACTT | TTGCTGGTTGAGGTCCGCTAGGATCC |
| p21 | GCAGACCAGCATGACAGATTT | GGATTAGGGCTTCCTCTTGGA |
| p27 | GGTTAGCGGAGCAATGCG | TCCACAGAACCGGCATTTG |
| bak1 | GGTCCTGCTCAACTCTACCC | CCTGAGAGTCCAACTGCAAA |
| bax | GACCGGAAAGTGCGACACA | GCTGACAGGTTCCACAAAGGT |
| bclxl | GATCCCCATGGCAGCAGTAAAGCAAG | CCCCATCCCGGAAGAGTTCATTCACT |
| GAPDH | CACCCAGAAGACTGTGGATGG | GTCTACATGGCAACTGTGAGG |

**Table S5. Primer sequences for** constructing plasmids

| **Plasmid** |  |
| --- | --- |
| Sp1-1 mutant | F: GACGCGTGACCGTGGCTGACCCCACCAGTCTTCGCGGGCTTC |
|  | R: GGTGGGGTCAGCCACGGTCACGCGTCCGCACCCCTGC |
| Sp1-2 mutant | F: GCGCCCCGCGCTGACGCCGCCGCCGCCGCCGACACCAC; |
|  | R: GCGGCGGCGGCGTCAGCGCGGGGCGCGAGGAACCGGGCGC |
| Sp1-3 mutant | F: CAGCTCGGCTCCAGAGCTGCCTGGCTGGCGTCACGGC; |
|  | R: GCCAGCCAGGCAGCTCTGGAGCCGAGCTGACGCCGGCGGC |
| Sp1-4 mutant | F: CACGGCCCGGCCCAGCCCCTCCTTCCCCTCCCCCGC; |
|  | R: GGAAGGAGGGGCGTGGGCCGGGCCGTGA CGCCAGCCAG |
| p27promoter 1-Luci | F: ACTGACTCGAGTCCAATAAATTGACAGCCTTAAAAGTAG |
|  | R: ACTGA ACGCGT TCCCTCAATGAAAATAAGAAAGGAACT |
| p27promoter 2-Luci | F: ACTGACTCGAGTGATAAGTGCCGCGTCTACTCC |
|  | R: ACTGAACGCGTGTGGCAAAGCCCGTCCGAGT |
| Klf4promoter 1-Luci | F: ACGATACGCGTGAACCCAGGGAGCCGACAAT |
|  | R: ACGATAGATCTACGAAGCCAAAACCCAAAAC |
| Klf4promoter 2-Luci | F: ACGATACGCGTTTTGGGTTTTGGCTTCGTTT |
|  | R: ACGATAGATCTTCAGTGGTGGTCCCCTGTTG |

**Supplemental Figure Legends**

**Figure S1. VPA significantly inhibits cell proliferation through cell cycle arrest in human leukemia cells.**

1. Cell proliferation assays of VPA treatment at different concentrations (0.5 mM, 1.0 mM, and 2.0 mM) in K562, HL-60, and U937 cells, respectively. *, **, and *** indicate *p* < 0.05, *p* < 0.01, and *p* < 0.001, respectively.
2. Cell cycle assays of VPA treatment at different concentrations (0.5 mM, 1.0 mM, and 2.0 mM) in K562, HL-60, and U937 cells, respectively. *, **, and *** indicate *p* < 0.05, *p* < 0.01, and *p* < 0.001, respectively.
3. QRT-PCR assays showing the effects of VPA (0.5 mM, 1.0 mM, and 2.0 mM) on the expression level of proliferation-associated genes (p21, p27, bak1, bax, and bclxl) in K562 cells. **, *** indicate *p* < 0.01, *p* < 0.001, respectively.
4. Western blotting assays showing the effects of VPA (0.5 mM, 1.0 mM, and 2.0 mM) on expression levels of p21 and p27 in K562 cells. Ctrl, cells infected with viruses carrying the empty vector. GAPDH was used as the loading control.

**Figure S2. Mocetinostat but not Tubastatin A inhibits cell proliferation through cell cycle arrest in human leukemia cells.**

1. Cell proliferation assays of Mocetinostat and Tubastatin A treatment at different concentrations in K562 cells. *, **, and *** indicate *p* < 0.05, *p* < 0.01, and *p* < 0.001, respectively.
2. Cell cycle assays of Mocetinostat and Tubastatin A treatment at different concentrations in K562 cells. *, **, and *** indicate *p* < 0.05, *p* < 0.01, and *p* < 0.001, respectively.
3. QRT-PCR assays for the effects of Mocetinostat and Tubastatin A treatment at different concentrations on the expression levels of Klf4, p21 and p27 in K562 cells. *, **, and *** indicate *p* < 0.05, *p* < 0.01, and *p* < 0.001, respectively.
4. Western blotting assays for the effects of Mocetinostat and Tubastatin A on expression levels of Klf4, p21 and p27 in K562 cells. Ctrl, cells treated with 0 mM of Mocetinostat and Tubastatin A. GAPDH was used as the loading control.

**Figure S3.** **Other members of Class I** **HDACs exhibit a different effect on leukemia cells.**

1. Western blotting analyses for the knockdown efficiency of HDAC2, HDAC3, and HDAC8 in K562 cells after infected with lentiviruses containing the corresponding shRNA (shHDAC2, shHDAC3, shHDAC8). GAPDH serves as a loading control.
2. The CCK8 assays for the effects of shHDAC2, shHDAC3, shHDAC8 on cell proliferation in K562 cells at day 1, day 2, day 3, and day 4, respectively. **, *** indicate *p* < 0.01, *p* < 0.001, respectively.
3. FACS analyses for the effects of shHDAC2, shHDAC3, shHDAC8 on the cell cycle in K562 cells. *, *** indicate *p* < 0.05, *p* < 0.001, respectively.
4. FACS analyses for the effects of shHDAC2, shHDAC3, shHDAC8 on the apoptotic rate in K562 cells.
5. Western blotting for the effects of shHDAC2, shHDAC3, shHDAC8 on the expression of Klf4, respectively.

**Figure S4. VPA significantly increases the Klf4 expression and histone acetylation level at its promoter region in human leukemia cells.**

1. QRT-PCR assays showing the effects of VPA treatment at different concentrations (0.5 mM, 1.0 mM, and 2.0 mM) on the expression levels of Klfs (Klf1, Klf2, Klf3, Klf4, Klf5, Klf6, Klf7, Klf8, Klf11, and Klf13) in K562 cells. *, **, and *** indicate *p* < 0.05, *p* < 0.01, and *p* < 0.001, respectively.
2. Western blotting assays showing the effects of VPA treatment at different concentrations (0.5 mM, 1.0 mM, and 2.0 mM) on the expression levels of Klfs (Klf1, Klf3, Klf4, and Klf7) in K562 cells. GAPDH was used as the internal control.
3. Sketch drawing of the 6 sites located at the *p21* and *p27* promoter, respectively.
4. Dual-luciferase reporter assay for the two fragments of p27 promoter activity in HEK 293T cells after Klf4 knockdown. *, ** indicate *p* < 0.05, *p* < 0.01, respectively.
5. Co-IP assays showing the interaction between HDAC1 and Klf4.
6. ChIP-PCR assays for acetylation levels of histone H3 and H4 at the *Klf4* promoter regions after treatment with VPA. *, ** indicate p < 0.05, p < 0.01, respectively.
7. Dual-luciferase reporter assay for the two fragments of Klf4 promoter activity in HEK 293T cells after HDAC1 knockdown. ** indicates *p* < 0.01.
8. Western blotting assay for the effects of Mocetinostat treatment on the expression levels of Sp1 at different concentrations (0.075 μM, 0.15 μM, and 0.5 μM) in K562 cells.
9. Western blotting assay for the effects of Mocetinostat treatment on the expression levels of total Sp1 and acetyl-Sp1 at different concentrations (0.075 μM, 0.15 μM, and 0.5 μM) in K562 cells.
10. ChIP-PCR assays showing the binding of Sp1 at the *Klf4* promoter regions after treatment with Mocetinostat. *, **, indicate p < 0.05, p < 0.01, respectively.
