# Supplementary figures and images for: HDAC1 and Klf4 interplay critically regulates human myeloid leukemia cell proliferation
Source: Cell Death Dis. 2014 Oct 23;5(10):e1491–. doi: 10.1038/cddis.2014.433 (PMC4237257; doi:10.1038/cddis.2014.433)

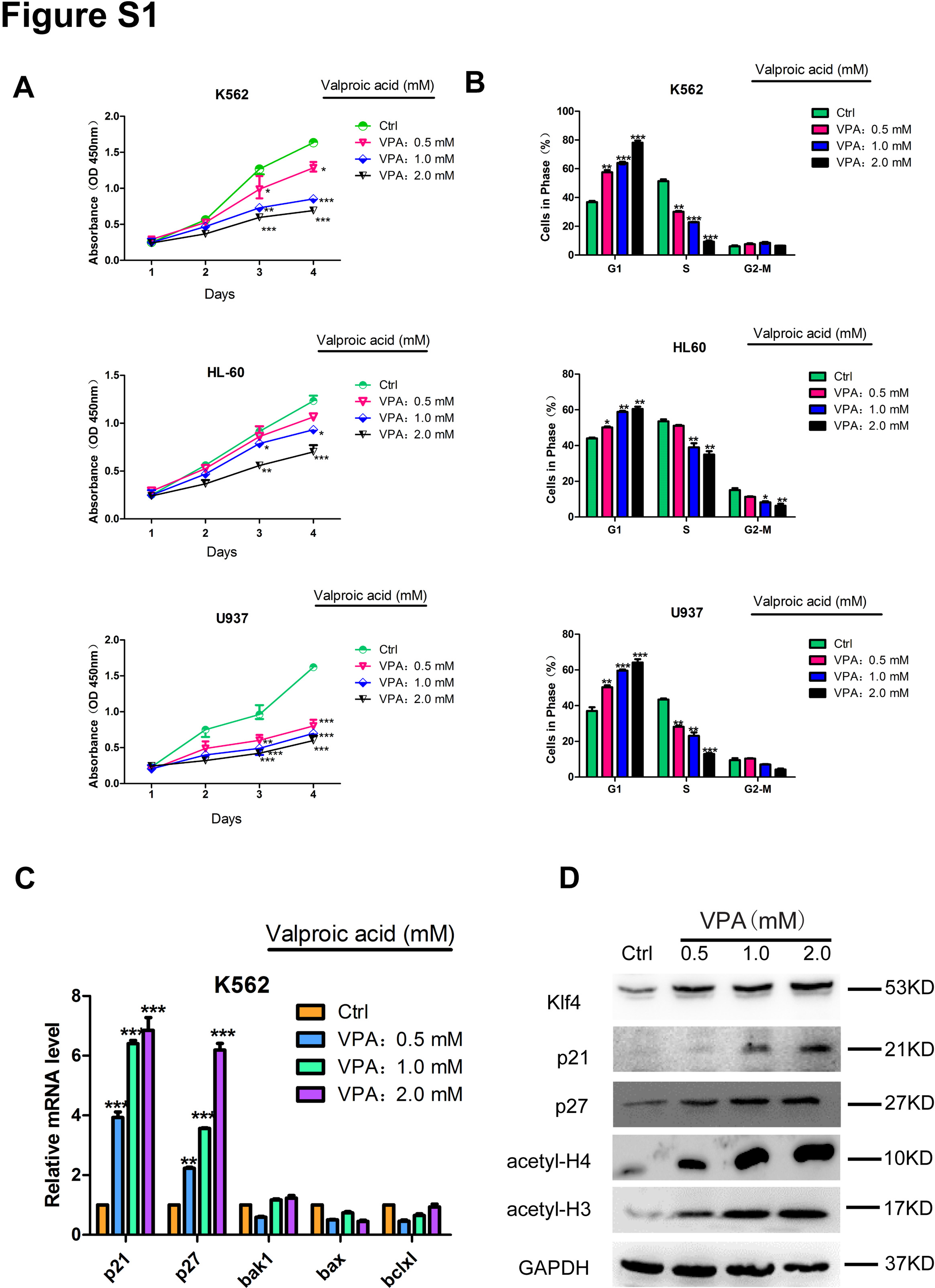

Supplement: Supplementary Figure S1 [file cddis2014433x2.tif]

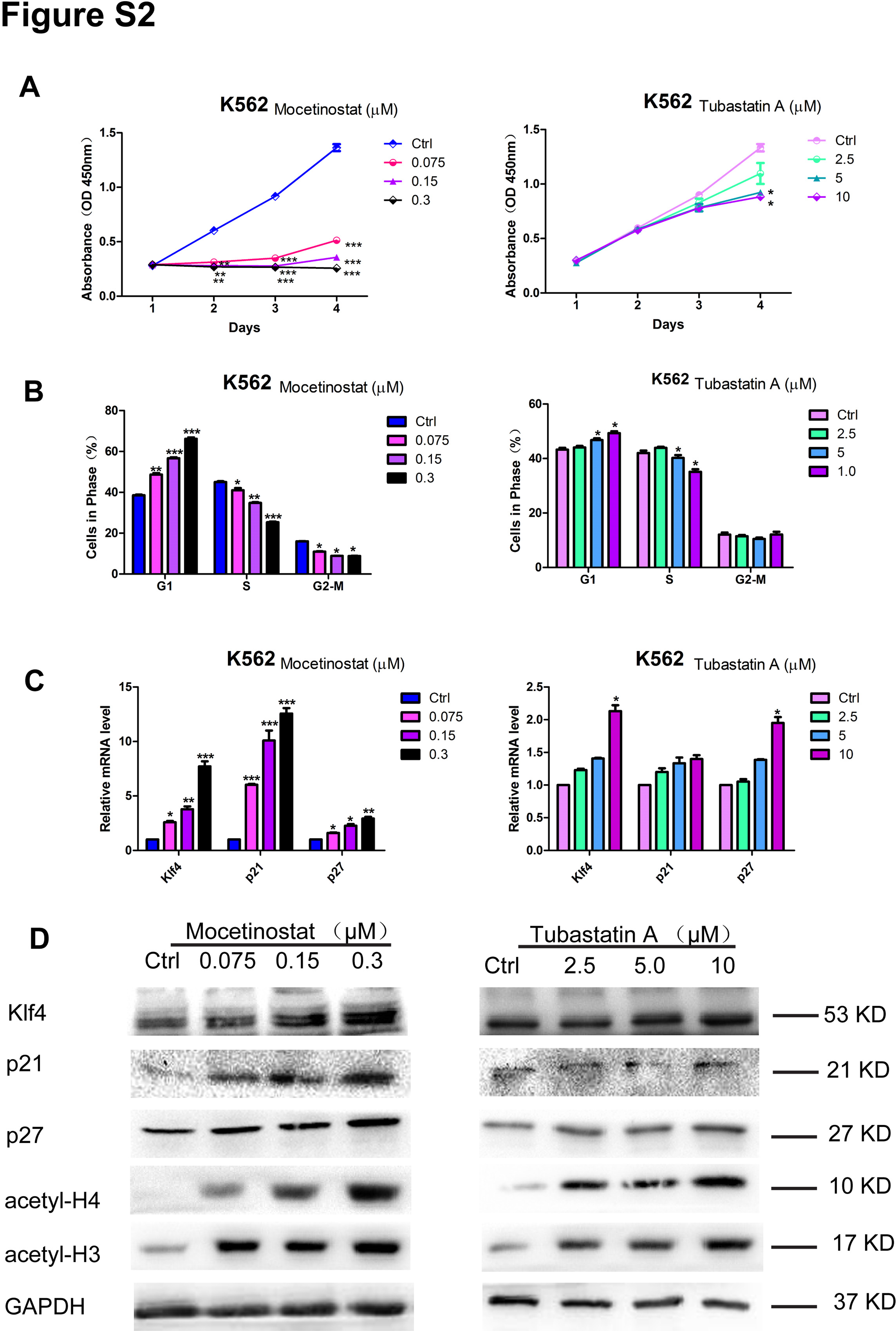

Supplement: Supplementary Figure S2 [file cddis2014433x3.tif]

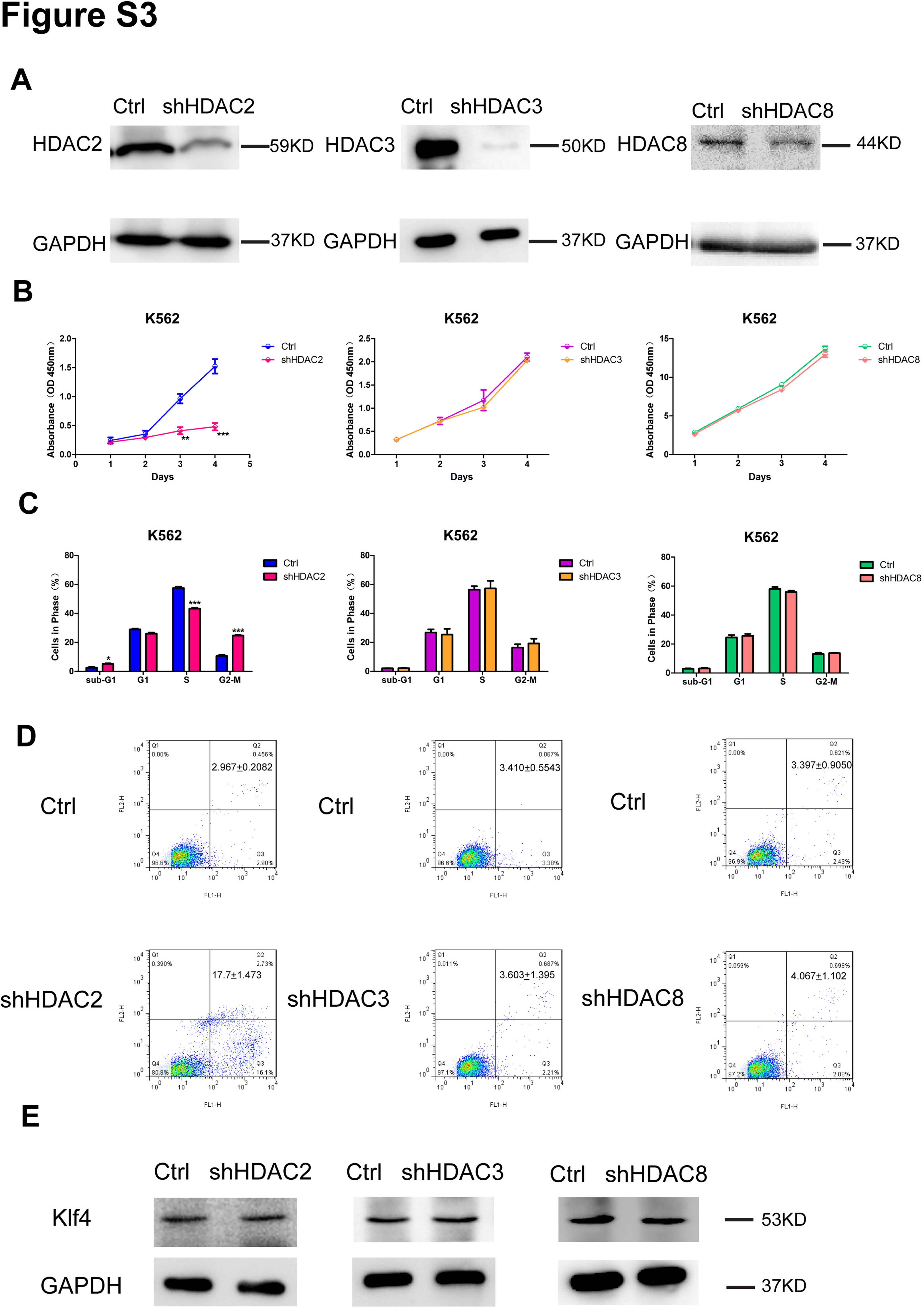

Supplement: Supplementary Figure S3 [file cddis2014433x4.tif]

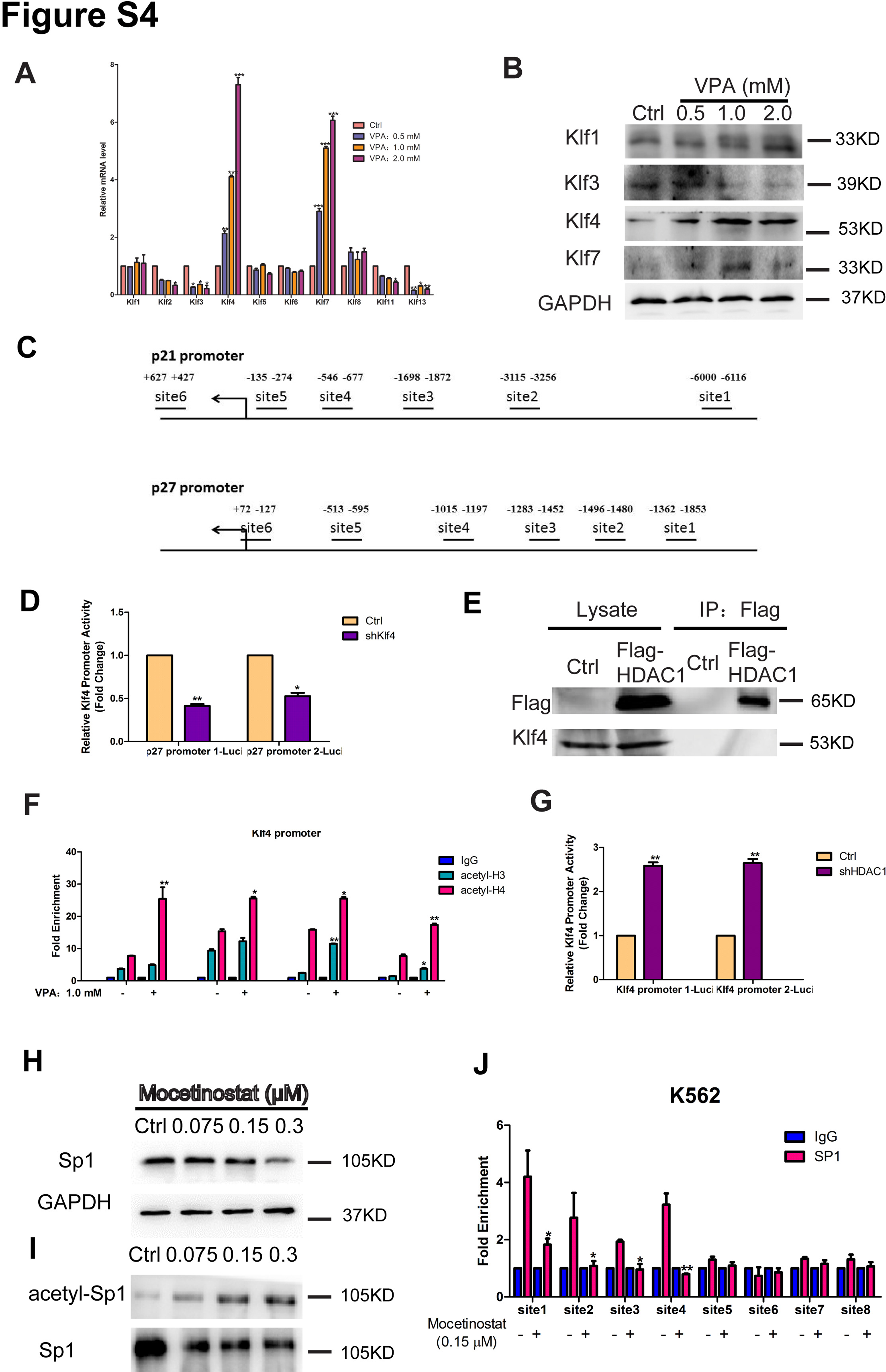

Supplement: Supplementary Figure S4 [file cddis2014433x5.tif]
